# Palliative care access: a matter of life and death

**DOI:** 10.1016/j.lanepe.2023.100586

**Published:** 2023-01-21

**Authors:** William E. Rosa, Meghan McDarby, Harvey M. Chochinov

**Affiliations:** a Department of Psychiatry and Behavioral Sciences, Memorial Sloan Kettering Cancer Center, New York, USA; b Department of Psychiatry, Max Rady College of Medicine, University of Manitoba, Manitoba, Canada; c CancerCare Manitoba Research Institute, Manitoba, Canada

More than 61 million people worldwide experience serious health-related suffering that could be relieved with palliative care (PC)^1^—a person- and family-centered approach to alleviating physical, psychological, social, and spiritual distress. Nafilyan and colleagues^2^ found an association between severe physical health condition diagnosis and higher suicide risk that underscores the well-established need for PC access as a component of universal health coverage.^3^ PC integration is recommended at the time of serious illness diagnosis in conjunction with curative treatment to optimize quality of life outcomes.^1^^,^^3^

Nafilyan et al.^2^ call for combined physical and mental health support. However, there are several PC implications to consider (Panel). Building on health systems' existing PC infrastructure to provide whole-person interventions—inclusive of mental health care—is a resource-conscious, cost-effective approach^1^^,^^3^ to better assess individual needs and discriminate between depression, suicidality, and adjustment to illness. Furthermore, health professionals trained in primary PC^4^ (e.g., generalist level skills) can help identify critical caregiver and community-based social supports while clarifying patients’ concerns and anticipating potential existential crises (Panel). PC also provides a model that can operationalize short-term therapies (e.g., dignity therapy)^5^ in rapidly changing clinical circumstances, especially for patients diagnosed with advanced or end-stage disease.

**Panel: Implications for palliative care provision starting at serious illness diagnosis**

• Beneficiaries of PC include patients, caregivers, families and social support networks, surrogate decision-makers, communities, and the broader health team at all levels of care, strengthening interprofessional and cross-team communication and promoting transitional care needs.
• PC starting at diagnosis can foster clinician–patient relationships through iterative goals of care conversations driven by patient and family core values, establishing trust early in the disease process to assist later during high-stakes scenarios, care transitions, and ethical dilemmas.
• A PC lens at diagnosis can assist health professionals to better learn the patient narrative and prioritize personhood as the central focus of the care plan.
• A holistic PC approach can help to address the overall burden of suffering that informs distress and decision-making (e.g., the choice to end one's life), including physical, psychological and/or psychiatric, social, cultural, spiritual, existential, legal, and/or ethical factors.
• All health professionals should be equipped with primary PC skills, including basic pain and symptom management, psychological and spiritual screening, social assessment and social prescribing, and early identification of the need for specialty PC involvement.
• Specialty PC (where available) can be consulted starting at diagnosis to assist with complex or refractory pain and symptom management; psychiatric emergencies; complicated social or family dynamics; spiritual or existential crises; and/or cultural, ethical, or legal disagreements.
• Specialty PC should be considered at the time of diagnosis any time a patient expresses a desire for hastened death; these inquiries need additional expert exploration to ensure optimal pain and symptom management, appropriate connection to resources, and tailored care plans with close monitoring.

The determinants of suffering are varied and subjective; its alleviation requires holistic, concurrent attention to myriad domains of care. PC provision is a moral obligation for all health professionals to protect public safety and wellbeing amid serious illness diagnosis and the heightened risk of suicide. Indeed, it is a matter of life and death.

## Contributors

WER: Conceptualization, Writing—Original Draft, Writing—Review & Editing; MM: Writing—Review & Editing; HMC: Writing—Review & Editing.

## Declaration of interests

WER has received funding unrelated to this manuscript from 10.13039/100015348Cambia Health Foundation, 10.13039/100000867Robert Wood Johnson Foundation, and 10.13039/100017234The Rita and Alex Hillman Foundation. MM is funded by 10.13039/100000054NCI/10.13039/100000002NIH award number T32CA00946. WER and MM acknowledge 10.13039/100000054NCI/10.13039/100000002NIH award number P30CA008748. Unrelated to this manuscript, HMC has received grant funding from 10.13039/100008794Research Manitoba, 10.13039/100009395CancerCare Manitoba Foundation, 10.13039/501100000024Canadian Institutes of Health Research; receives book royalties from Oxford University Press; has received payment or honoraria from the Societa Italianá Di Cure Palliative National and Saskatoon Hospice and Palliative Care Association; and serves on the ReSet Pharma/NYU advisory board for psilocybin protocol development in advanced cancer.
